# How vision and self-motion combine or compete during path reproduction changes with age

**DOI:** 10.1038/srep29163

**Published:** 2016-07-06

**Authors:** Karin Petrini, Andrea Caradonna, Celia Foster, Neil Burgess, Marko Nardini

**Affiliations:** 1 Department of Psychology, University of Bath, UK; 2 UCL Institute of Ophthalmology, London, UK; 3 UCL Research Department of Neuroscience, Physiology and Pharmacology, UK; 4 Centre for Integrative Neuroscience, University of Tübingen, Germany; 5 UCL Institute of Cognitive Neuroscience, London, UK; 6 University College London Institute of Neurology, UK; 7 Department of Psychology, Durham University, UK

## Abstract

Human adults can optimally integrate visual and non-visual self-motion cues when navigating, while children up to 8 years old cannot. Whether older children can is unknown, limiting our understanding of how our internal multisensory representation of space develops. Eighteen adults and fifteen 10- to 11-year-old children were guided along a two-legged path in darkness (self-motion only), in a virtual room (visual + self-motion), or were shown a pre-recorded walk in the virtual room while standing still (visual only). Participants then reproduced the path in darkness. We obtained a measure of the dispersion of the end-points (variable error) and of their distances from the correct end point (constant error). Only children reduced their variable error when recalling the path in the visual + self-motion condition, indicating combination of these cues. Adults showed a constant error for the combined condition intermediate to those for single cues, indicative of cue competition, which may explain the lack of near-optimal integration in this group. This suggests that later in childhood humans can gain from optimally integrating spatial cues even when in the same situation these are kept separate in adulthood.

Humans and other animals use a range of spatial strategies and cues (e.g., self-motion and visual landmarks) to move and find locations in the enviroment^1^,^2^,^3^,^4^,^5^. Developmental studies with humans have documented major changes in spatial cognition and navigation abilities from infancy through to adulthood^6^. For example, although there is evidence that children as young as 3 years can use spatial representations both based on the body (i.e., egocentric representations) and on the enviroment (i.e., allocentric representations) when recalling the location of an hidden toy^7^,^8^, abilities to combine multiple spatial representations to improve precision have not been found until after 8 years^9^. Specifically, Nardini, *et al*.^9^ showed that adult participants increased their precision (reduced their variability) at relocating an object by near-optimally integrating^10^,^11^ visual landmarks and self-motion cues when both were available (for a replication of these results in young and old adults see also)^12^. However, in Nardini, *et al*.^9^, children up to 7–8 years old did not use this kind of near-optimal integration, and instead switched between cues, relying either on self-motion or on visual landmarks when relocating the object.

Several studies e.g.^13^,^14^,^15^,^16^, have now shown that this late maturation of optimal multisensory integration is not specific to spatial navigation, but extends to many other spatial and non-spatial tasks (e.g., object size discrimination) and combinations of cues (e.g., vision and touch or sound and touch). Taken together, these results suggest that in the spatial domain, even once multiple individual kinds of spatial representations become available in childhood^6^, they may not be immediately combined with each other to guide behaviour. What is not clear yet is why the ability to combine different spatial representations and sensory cues optimally develops this late in childhood. One prominent theory proposed to explain this late maturation of optimal cue combination is that during childhood, depending on the task, one of the senses is used as the benchmark for recalibrating the others^13^,^17^,^18^. Hence, in spatial tasks, visual information is used to calibrate other modalities. Further evidence in favour of the proposal that children use visual information to calibrate other sensory cues in spatial tasks comes from findings that, compared with adults, children have substantial difficulties discounting or ignoring irrelevant visual information in multimodal tasks^19^,^20^,^21^. Children’s difficulties in discounting or ignoring visual information even when it is irrelevant are consistent with the possibility that they rely heavily on visual information, e.g. to calibrate other sensory cues.

Although in some experimental situations^9^,^12^,^22^ adult participants show cue combination, in others they do not^23^,^24^. For example, experiment 1 of Tcheang, *et al*.^25^ showed, using a triangle completion task, that after being presented with visually conflicting information when walking along an outbound path, adult participants used a representation combining visual and self-motion cues when finding their way back to their start point in darkness. However, there was no decrease in the variance of responses when both visual and self-motion cues were available on the outbound path compared to when the outbound path was walked in darkness, even when both types of cue were consistent. This indicates that adult participants did not show the potential benefits of optimal combination of vision with self-motion. Another example of lack of integration in adult participants comes from studies of multisensory spatial movements (e.g., rhythmic movements of the upper arm), showing that visual information is more accurate and thus dominates the proprioceptive^24^.

Zhao and Warren^23^ have made a recent proposal that may explain seeming inconsistencies in findings from these studies. Zhao and Warren^23^ suggest that while the evidence in support of optimal cue integration comes from measurements of response variability or precision^9^, those for cue dominance or competition (i.e., greater reliance on one cue) come from measurements of response accuracy e.g.^25^. Zhao and Warren^23^ show evidence in favour of this in a homing study (object relocation task, similar to that of Nardini, *et al*.)^9^. They measured both response variability (how tightly clustered their chosen object relocation positions were) and accuracy (how close the clustered chosen positions were to the correct position) and showed that while adult participants behaved according to optimal cue combination in their homing variability response, they behaved according to cue competition in their homing accuracy response.

These recent results, and the others described above, raise a series of interesting questions. First of all, do adults use different multisensory models (cue integration and cue competition) for variability and accuracy of responses at all times, or are these models used flexibly depending on the situation? For example, would results be different to those of Zhao and Warren^23^ in a design similar to that of Tcheang, *et al*.^25^, where adults appeared not to benefit from redundant visual information? Would children (old enough to show cue integration) show a similar or different use of these multisensory models compared to adults, due to their previously shown stronger reliance on visual information during spatial tasks^14^?

We tested a group of 10- to 11-year-old children and adults in a path reproduction task in darkness, using immersive virtual reality to decouple visual and self-motion information when walking along a path. Both child and adult participants walked a two-legged path under a self-motion only condition (by walking in darkness), a visual + self-motion condition (by walking in the virtual room), and a visual only condition (watching a pre-recorded walk in the virtual room while standing still). They then reproduced the same path in darkness. We analysed both variability and accuracy. If participants remember a multisensory representation of the path that integrates visual and self-motion cues, then participants’ variability should decrease and accuracy should increase during the subsequent path reproduction in darkness in the combined visual + self-motion condition compared to the visual or self-motion conditions.

## Method

### Participants

Participants were fifteen 10- to 11-year-olds (7 females and 8 males, mean age of 10.3, SD of 0.4) and eighteen adults (7 females and 11 males, mean age of 25.5, SD of 5.6). Participants, recruited by leaflets and press advertisements, were from a socially and ethnically diverse population in central London, UK. All experiments were performed in accordance with relevant guidelines and regulations. Adults and children’s parents or guardians gave informed consent to participate, and the study received ethical approval from the research ethics board at University College London.

### Apparatus

**Figure 1:**
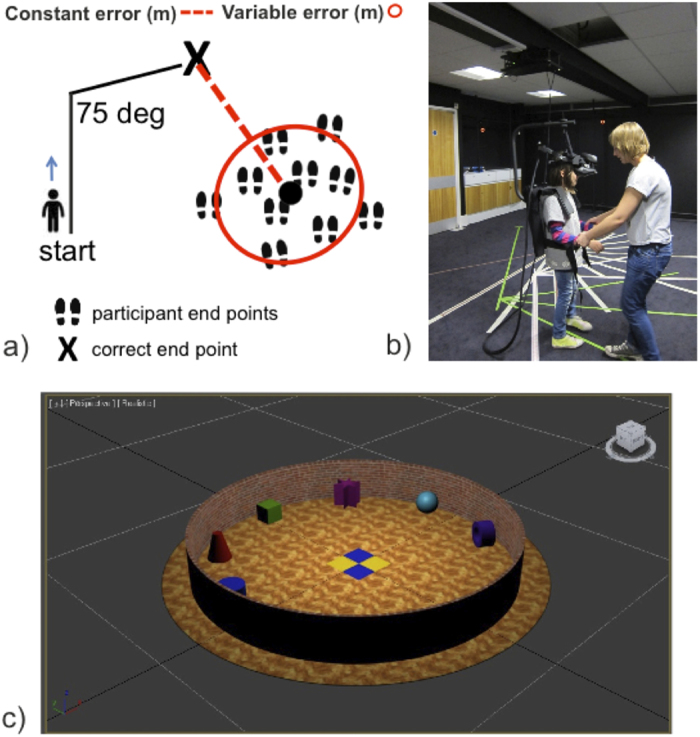
Experimental set up and task/estimates description. (**a**) Participants were guided along a two-legged path either in darkness (self-motion condition), or were guided while able to see the virtual room (visual + self-motion condition), or remained stationary while they viewed a pre-recorded video of walking the path in the virtual room (visual condition). After being guided back to the start point in darkness (in the self-motion or visual + self-motion condition) or reaching the end of the pre-recorded video (in the visual condition) they attempted to reproduce the path in darkness. The trajectories and points at which participants stopped at the end of each trial were recorded, and measures of variable error and constant error were obtained for each participant. (**b**) A child wearing the apparatus used to reduce the HMD weight on the head by redistributing it to the shoulders. (**c**) The virtual room used in the experiment. Participants were positioned at the opposite side of the circular arena with respect to the green cube, at the start of every trial. However, they changed position in the real room on every trial.

Figure 1a illustrates the two-legged path that participants were asked to reproduce. The first leg of the path was 2.7 meters in length, while the second leg 2 meters in length. The turning angle between them was 75 degrees, and was selected after running a pilot experiment with 6 adult participants and three different turning angles of 45, 75 and 105 degrees. Four two-legged paths were marked on the floor of the real room using tape of four different colours (see Fig. 1b). The four paths had different start points in the real room (although participants were always facing the green cube in the virtual room at the start, Fig. 1c). We used 4 starting points in the real room to avoid participants using the distance to the physical starting point (from the point they stopped at) to correct their previous response^25^. Two of the four paths had a turn to the left, while the other two had a turn to the right, in order to represent both turning directions at two different starting positions. The experiment took place in a room (6.5 m × 7.75 m) with black walls and black carpet, hosting a virtual reality system composed of an optical tracking system (8 Vicon Bonita MX13 cameras; Vicon, Oxford, UK) and a head mounted display (HMD) with a wide (111 degree) field of view (nVisor SX111; NVIS, Reston, VA). The camera system had five HMD-mounted reflective markers, which tracked participants’ positions. The 3D position coordinates for the markers were saved using Vizard software (4.10.0005; WorldViz LLC, Santa Barbara, CA), and were additionally used to update in real-time the participant’s position in the virtual room, which was also generated and presented using Vizard. This was achieved with minimum latency thus giving a realistic immersive experience, as confirmed by participants’ reports. The virtual room shown through the HMD was a circular arena (very similar to the one originally used in Tcheang, *et al*.)^25^, designed using Autodesk 3ds Max 2012 × 64. The virtual space was a circular room 6 meters in diameter containing seven large objects around its perimeter, with a parquet floor, 2-metre high brick wall, and yellow and blue tiles indicating the center (Fig. 1c).

### Procedure

Participants and their parents (in the case of children) were received in the waiting room just outside the virtual reality lab by two experimenters, they were welcomed and the task was explained. After participants received the study information and gave their consent to participate, the experimenters measured their interpupillary distance (IPD). This measure was used to set the rendering of the virtual environment appropriately for correct stereoscopic depth perception for each participant during the study. The experimenters also asked participants to wear a pair of foam earplugs and to close their eyes until the experimenter asked them to open them again. This was done to prevent participants from using any background noise and/or the real room layout and floor markings to orient themselves during the task. Then one of the experimenters guided the participant inside the real room and the two experimenters positioned the HMD on the participant’s head, ensuring that it was well fitted (i.e., the participant could not see anything outside the display) and comfortable. For child participants the experimenter also fitted a harness over the participant’s shoulders. This harness was connected to the top of the HMD and in order to make the headset lighter on participants’ heads and make navigation easier (see Fig. 1b). After fitting the HMD the experimenters displayed the virtual room (Fig. 1c) using the HMD and asked participants to open their eyes and start looking and walking around to familiarize themselves with the virtual room. Once participants reported feeling comfortable and not seeing anything outside the headset, the experiment started. One of the experimenters guided participants to one of the 4 starting points in the real room and participants carried out the task in a two- or three-trial practice session. As the task was easy to understand this number of practice trials was sufficient, as demonstrated by all the child and adult participants understanding and performing the task properly within this number of practice trials. The order of the 4 starting points was randomized for both the practice trials and the main experiment. For each trial the number corresponding to the selected starting point was shown on the computer screen for the experimenter to see. The main experiment consisted of 30 trials, 10 trials for each condition: visual only (V), self-motion only (SM), and visual + self-motion (V + SM). The order of the three conditions was counterbalanced across participants.

### Condition SM

On each trial participants were positioned at the selected start point and were then guided by the experimenter along the two-legged path, in darkness, while wearing the HMD. The experimenter informed the participant when to start walking and when to stop. When participants reached the end of the two-legged path, the experimenter took them back to the original start point following a variable and irregular path. Once back at the start point participants were asked to reproduce the path in darkness and stop when they thought that they had reached the original end point.

### Condition V

On each trial participants were positioned at the selected start point and were asked to stand still while wearing the HMD. In this condition participants did not walk but watched a pre-recorded walk of the path in the virtual room. The pre-recorded path was obtained by asking one average-height child and adult to walk along each of the four paths in the real room (all presented as one path in the virtual room). The average height for the examined age range was 1.70 m (average between female and male average heights) for adults and 1.40 m for 10- to 11-year-old children as reported by the National Institute for Care and Health Excellence^26^. The child and adult who provided these recordings did not participate in the study. The experimenter informed the participant when the recording started and ended. When the recording reached the end (i.e., the participant reached the end of the two-legged path), participants clicked a wireless mouse, which blacked out the virtual room and put participants in darkness. Then participants were asked to reproduce the path in darkness and stop when they thought that they had reached the original end point.

### Condition V + SM

On each trial participants were positioned at the selected start point and were then guided by the experimenter along the two-legged path, while wearing the HMD and seeing the virtual room. The experimenter told the participant when to start walking and when to stop. When participants reached the end of the two-legged path they clicked a wireless mouse, which blacked out the virtual room and put them in darkness. The experimenter then took them back to the original start point following a variable irregular path. Once back at the start point participants were asked to reproduce the path, in darkness, and stop when they thought that they had reached the original end point.

A break of 10–15 minutes was taken after each condition, or when needed. Each time participants exited the real room, before ending the experiment, they were asked to close their eyes while they were guided to the waiting room. After each break the same initial procedure, from entering the real room to fitting the HMD, was repeated before starting the next condition. Finally, the experimenters took off the HMD and participants could see the real room, be debriefed and answer a few final questions. When asked, all participants reported to be disoriented and not to know whether they were repositioned in the same place before reproducing the path. No participant reported knowing that there were multiple (four) start points. Furthermore, the majority of participants were very surprised when seeing the real room at the end and reported that they were expecting a much larger, circular room (i.e., one like the virtual room). Finally, they reported that they felt as if they needed to stop themselves from walking during the visual only condition, indicating that they felt that they were the person walking in the recording.

## Results

A bivariate normal distribution was fitted to each participant’s end positions, relative to the correct end-point (i.e., where participants decided to stop, see Fig. 1a and Supplementary Figure S1), to estimate the x mean, y mean, x variance, y variance and x-y covariance for each condition. The FASTMCD algorithm^27^, as implemented in the Libra toolbox for Matlab^28^, was used to estimate these values robustly, with the assumption of 1% aberrant (outlier) values (i.e. a value of 0.99 for the alpha parameter). The sum of the variance in x and y directions was used to obtain a single measure of total variable error, reflecting the uncertainty of spatial estimates (area of the ellipses in Figure S1). The optimal predicted estimate for variance in the V + SM condition was calculated by entering the V and SM obtained measures of variable error into the Bayesian maximum likelihood estimation (MLE) model^10^,^29^. This predicts the combined-cue variance expected for a participant who combines single-cue estimates using reliability-weighted averaging. Additionally, a measure of constant error was calculated as the distance between the correct end location and the participant’s end position (distances between the green dot and the dot representing the centre of the ellipse in Supplementary Figure S1).

**Figure 2:**
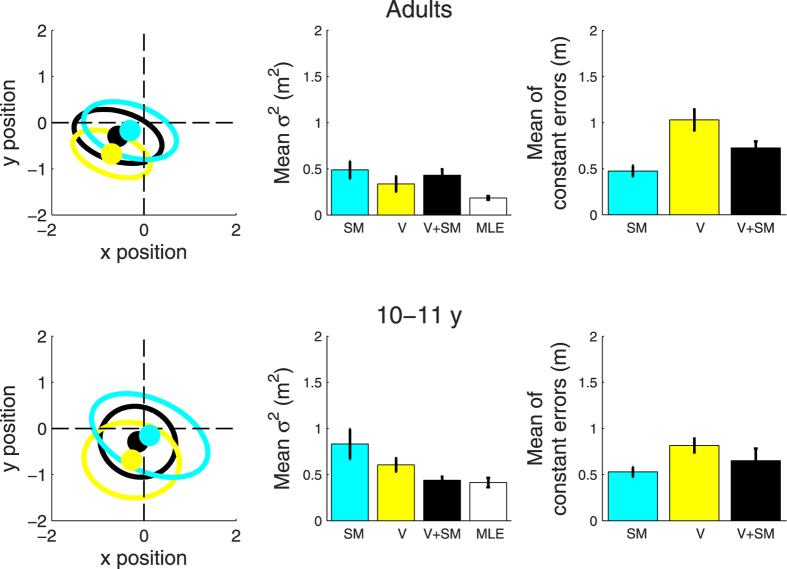
Average variable and constant error for child and adult participants. Average results for the child and adult participants performing the self-motion (SM and cyan), visual (V and yellow) and visual + self-motion condition (V + SM and black). The panels on the left represent the bivariate fitting (ellipses) and its centre (constant error of the mean), the panels in the centre represent the mean variable error (area of the ellipses), while the panels on the right represent the mean of constant errors (which is similar, but not equal to the constant error of the mean). Children’s results are shown in the bottom panels, while adults’ data are presented in the top panels. The bar labelled MLE (in white) in the central panels refers to the reduction in variability predicted by the maximum likelihood estimation model for the visual + self-motion condition. The predicted estimate (σ_*V*+*SM*_) was calculated individually for each subject, and then averaged, by entering the individual visual (σ_*V*_) and self-motion (*σ*_*SM*_) measure of total variable error into the equation 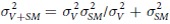. Error bars represent the standard error of the mean.

Figure 2 plots the average variable and constant errors for 10- to 11-year-old and adult participants. To test whether on average children and adults were less variable when given both visual and self-motion information (condition V + SM) than only visual or self-motion information (conditions V and SM), as predicted by cue combination, we carried out a series of planned one-tailed paired t-tests. These showed that total variance for the V + SM condition in children was significantly lower than both that for the V condition (*t*(14) = −2.031, *p* = 0.031) and the SM condition (*t*(14) = −2.383, *p* = 0.016). In addition, no difference was found between measured variance in the V + SM condition and that predicted by the MLE model (*t*(14) = 0.482, *p* = 0.638), indicating that reliability-weighted averaging of sensory cues was a good predictor of children’s performance in the multisensory V + SM condition. In contrast, adults did not show any difference in total variance between V + SM and either V or SM (V + SM vs. V: *t*(17) = 1.036, *p* = 0.156; V + SM vs. SM: *t*(17) = −0.497, *p* = 0.313), and showed a significant difference between measured V + SM variance and that predicted by the MLE model (*t*(17) = 3.937, *p* = 0.001); see Fig. 2. That is, adults’ performance was not well predicted by the cue combination model. Hence, while children reduced their variability by combining the two cues, adults did not.

Cue combination does not necessarily predict any reduction in constant error (systematic bias). Rather, relying partly on two cues with different constant errors –whether via cue averaging on each trial, as in the MLE model, or via use of one cue or the other on each trial, i.e., cue switching – predicts a constant error intermediate to those of the single cues. The ellipse plots of distributions of responses on the left in Fig. 2 show this general pattern: for both children and adults, centres of ellipses (dots) in the two-cue V + SM condition (black) are intermediate to those in V (yellow) and SM (cyan). Similarly, bar graphs on the right of Fig. 2 show average combined-cue (SM + V) constant errors to be intermediate to those with single cues V and SM in both age groups. For a statistical test of whether, on average, children and adults changed their constant errors given both visual and self-motion information (condition V + SM) as compared with either visual or self-motion alone, we carried out a series of planned one-tailed paired sample t-tests. These showed that mean absolute constant error for the V + SM condition was significantly lower than that for the V condition for the adult (*t*(17) = −2.730, *p* = 0.007) but not the child group (*t*(14) = −1.002, *p* = 0.166). Constant error for the V + SM condition was significantly greater than that for the SM condition for the adult (*t*(17) = 3.382, *p* = 0.002), but not the child group (*t*(14) = 0.896, *p* = 0.193). Both adults and children had a significantly smaller constant error for the SM condition than the V (adults: *t*(17) = 5.333, *p* < 0.001; children: *t*(14) = 2.675, *p* = 0.018); Fig. 2. In summary, in both groups, constant errors (biases) with combined cues tended to be intermediate to those of single cues, consistent with the use of both cues, whether via cue averaging or cue switching; see Fig. 2, ellipse plots. In adults, statistical comparisons also showed mean absolute constant errors with two cues to be intermediate to those with single cues.

Zhao and Warren (2015) showed that adult participants switched between visual and self-motion cues in their homing task when visual and self-motion cues were highly discrepant. In our study, the same switching strategy may explain adults’ performance. That is, the greater precision in adults’ V and SM responses could result in a larger separation between the distributions of responses indicated by either cue, leading them to switch between cues rather than combining them. This sort of breakdown of cue combination is expected under large conflicts^30^,^31^. To assess this possibility we first calculated, for each individual, the Mahalanobis distance between the distribution of V and SM end points by taking into account their variance. That is, for each individual, we calculated the Mahalanobis distance to each point (*x*_*V*_, *y*_*V*_) in the V distribution from the mean of the SM distribution(*Mx*_*SM*_, *My*_*SM*_) by dividing for the standard deviation of the SM distribution(*SDx*_*SM*_, *SDy*_*SM*_). Similarly, we calculated the Mahalanobis distance to each point (*x*_*SM*_, *y*_*SM*_)in the SM distribution from the mean of the V distribution (*Mx*_*V*_, *My*_*V*_) by dividing for the standard deviation of the V distribution(*SDx*_*V*_, *SDy*_*V*_). The following equations summarise this process:


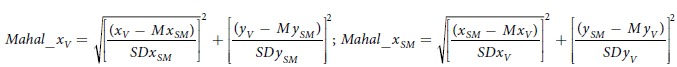


**Figure 3:**
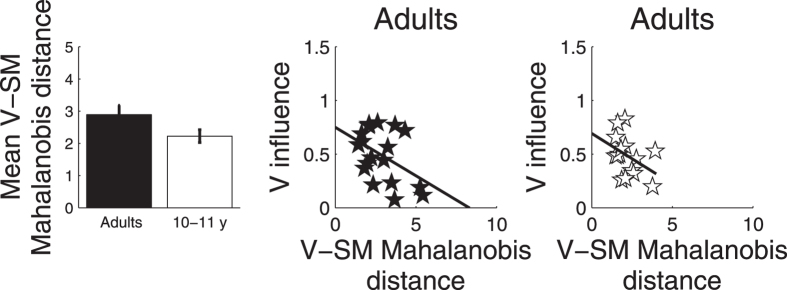
The left panel shows the Mahalanobis distance between V and SM distribution of end points, for the adult and child group. The central and right panels show the relation between the level of visual influence in the V + SM condition and the Mahalanobis distance for adults and 10- to 11-year-old children.

To obtain one measure of Mahalanobis distance between the distribution of V and SM for each individual, we calculated the mean of these individual Mahalanobis values. In this way, we obtained a value of Mahalanobis distance between V and SM distribution of points for each adult and child. A planned one-tailed t-test showed that the resulting difference in Mahalanobis distance was significantly greater for adults than children (*t*(31) = 1.842, *p* = 0.037), that is, adults experience the cues as more discrepant than children, even when the level of variance was considered (Fig. 3, left panel). We next examined whether the individual level of discrepancy between V and SM (the individual Mahalanobis distance) correlated with the way the two spatial cues were used. To this end, we measured the relation between the Mahalanobis distance and level of visual influence in V + SM responses (a measure of cue switching). Visual influence was calculated as the distance between mean V + SM and SM end points (*d*_*SM*_) divided by the sum of distances between V + SM and SM (*d*_*SM*_) and between V + SM and V (*d*_*V*_) end points, i.e., 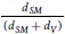. A directional (one-tailed) Pearson’ s correlation analysis revealed that within the adult group, the level of visual influence in the V + SM condition decreased significantly with the increase in Mahalanobis distance ( = −0.448, *p* = 0.031, Fig. 3 central panel). This relation did not reach significance in the child group, though this group showed a similar tendency to decrease the level of visual influence in the combined condition as the Mahalanobis distance increased (*r* = −0.0390, *p* = 0.075, Fig. 3 right panel).

**Figure 4:**
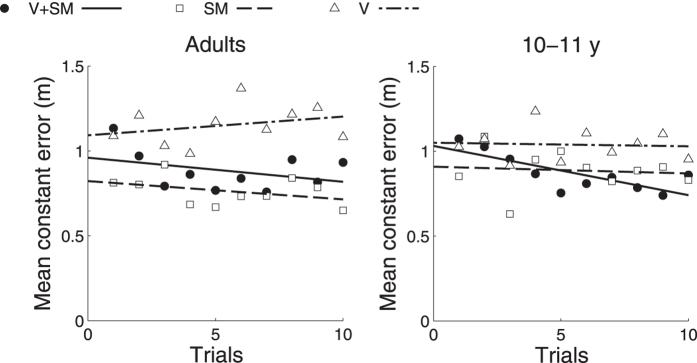
Relation between average constant error and number of trials for child and adult participants. Relation between the average constant error in visual-self-motion (V + SM), visual (V) and self-motion condition (SM) and the number of trials is presented separately for the adult (left panel) and child group (right panel).

Finally, we examined whether there was any learning effect in children and adults across the 10 trials, in each condition. As we obtained ten measures of constant error for each individual, we used regression to ask whether, on average, absolute constant error decreased with practice, even though there was no feedback. In contrast to adults, who did not show any significant correlation between trial number and size of mean constant error in the V, SM and V + SM conditions (Fig. 4; all *r* ≤ 0.483, all *p* ≥ 0.157), children showed a significant negative correlation between trial number and V + SM constant error (*r* = −0.795, *p* = 0.006). This suggests that children changed how they combined the two spatial cues over the course of the experiment, while adults did not. The reduction in children’s constant error over the course of the experiment (Fig. 4) would be consistent with initially relying more on the cue with higher constant error (vision; V) and later more on self-motion (SM). Another possible contributor to changes in constant error would be adaptation or recalibration of single cues. However, as there were no changes in constant error in either single cue condition, the effect is more likely due to changes in weighting for the two cues during combined-cue trials.

### Control analyses

In addition to the main analysis we carried out some control analyses. First of all, we examined whether the difference in height between the templates used during the pre-recording for the V condition and the child and adult participants affected the data. To test for this, we examined whether there was any relation between the difference in template-participant height and the length walked by each participant for the first and second leg of the path by running a series of Pearson’s correlation tests (see Supplementary Figure S2). Additionally, we tested whether the difference in template-participant height correlated with the measures of turning angle and constant error in the V condition. No significant correlation was found between the difference in template-participant height and these measures for either adults (all *p* ≥ 0.650) or children (all *p* ≥ 0.645). Hence, the templates provided good simulations of participants’ walking patterns and minor discrepancies in height did not affect the data.

Next, we checked the extent to which there were over or underestimations of length and turning angle in the different sensory conditions, for adults and children. This was done to better understand how different components of the path contributed to the overall measures of variable and constant error. To this end, we examined whether the average length walked by participants for the first and second leg of the path differed significantly from the real length and whether the average turned angle differed from the real angle (see Supplementary Figure S3). One sample t-tests showed that both adults (V: *t*(17) = −6.529, *p* < 0.001; V + SM: *t*(17) = −5.413, *p* < 0.001) and children (V: *t*(14) = −6.674, *p* < 0.001; V + SM: *t*(14) = −2.626, *p* = 0.02) significantly underestimated the length for the V and V + SM conditions when walking the first leg of the path. Only adults significantly underestimated this length in the SM condition (adults: *t*(17) = −3.473, *p* = 0.003; 10-11y: *t*(14) = 1.122, *p* = 0.281). Both adults (V: *t*(17) = −6.833, *p* < 0.001; V + SM: *t*(17) = −4.664, *p* < 0.001; M: *t*(17) = −3.800, *p* = 0.001) and children (V: *t*(14) = −6.108, *p* < 0.001; V + SM: *t*(14) = −2.935, *p* = 0.011; M: *t*(17) = −2.191, *p* = 0.046) significantly underestimated the length for all three conditions when walking the second leg of the path. Neither adults (*t*(17) ≤ −1.980; *p* ≥ 0.064) nor children (*t*(17) ≤ −2.003; *p* ≥ 0.065) significantly under or overestimated the turning angle for the three different conditions. The similarities between child and adult results indicate that both groups were quite accurate in their estimation of turning angle, while they were much less so in their estimation of walking length. This is especially evident for the V condition in both groups (see Supplementary Figure S3). This systematic underestimation of visual distance in a virtual reality environment is well known e.g.^24^,^25^.

## Discussion

Using immersive virtual reality to decouple spatial visual information from participants’ self-motion, we tested a group of 10- to 11-year-old children and adults in a path reproduction task. Both child and adult participants walked a two-legged path under a self-motion only condition (by walking in darkness), a visual + self-motion condition (by walking in the virtual room), and a visual only condition (watching a pre-recorded walk in the virtual room while standing still). They then reproduced the same path in darkness. We obtained both measures of response variability (i.e., how tightly clustered positions at which participants chose to stop were) and response accuracy (i.e., how close the clustered chosen positions were, on average, to the correct position). We predicted that if near-optimal integration of visual and self-motion cues occurred when walking along the path, to form a multisensory map, then participants’ variability during path reproduction would reduce, despite the lack of visual information (i.e., darkness). We showed that while adults did not reduce their response variability by integrating visual and self-motion cues when walking along the two-legged path, 10- to 11-year-old children did. This allowed children to reduce their path reproduction variability in darkness as predicted by the MLE model^10^,^29^. However, in both adults and children the combined-cue constant errors were intermediate to those of single cues (as predicted if both cues were used to some extent). However, significant differences between the constant error for the combined condition and those for the single cues were only found in the adult group.

These are interesting results for several reasons. First of all, adults did not optimally integrate the visual and self-motion congruent cues to reduce their variability during path reproduction, which is at odds with many previous findings^9^,^22^,^23^, but not others^25^. One explanation of these inconsistent findings could reside in the methodological differences between some of these studies and the present. In the Nardini, *et al*.^9^ and Zhao and Warren^23^ studies, participants memorized a spatial location with both visual and self-motion cues available and then navigated back to the same location under different conditions, with only visual cues, only self-motion cues, or both. When adult participants memorized the position with both cues and then navigated back with both cues they showed reduced response variability as predicted by optimal integration. In Kalia, *et al*.^22^ participants memorized the information visually and through self-motion, but then had to use touch to indicate their response on a map. That is, neither the visual nor the self-motion cue was available during the navigation response. In the present study, in contrast, although participants memorized the path with both visual and self-motion cues, they always reproduced the path in darkness (i.e., only one cue was present at reproduction). Thus, our study resembles that of Tcheang, *et al*.^25^ where adult participants memorized the two-legged path with both visual and self-motion cues and then returned to the start in darkness (using self-motion only).

Like Tcheang, *et al*.^25^ did not find improved accuracy of the combined cues when compared to the self-motion single cue in adult participants, indeed in our study self-motion alone showed a lower constant error (i.e., less biased) than self-motion combined with visual information. This may be because only the self-motion information was present during both memorization and reproduction, while the visual information was only available at memorization. That is, participants could directly match self-motion during reproduction to the remembered self-motion, whereas reproduction information can only be indirectly compared to remembered visual information. This indirect comparison may utilize the generation of visual imagery from self-motion during reproduction (Tcheang *et al*.)^25^. The process of generating visual imagery from self-motion likely creates additional bias over and above that already present in the reproduced self-motion.

Intriguingly, 10- to 11-year-old children did near-optimally integrate visual and self-motion cues when walking the path, showing reduced variability during path reproduction in darkness. Although this is not the first time that children have been shown to integrate cues when adults do not^21^, or do less so^20^,^32^,^33^, it is the first time that children have been shown to obtain behavioural benefits from this where adults do not. An adaptive combination view^34^ can explain these results, if we accept that children and adults use different models to process the spatial cues, due to differences in their perceived validity and salience. During childhood the body grows and changes rapidly^16^, while at an adult age natural body changes are minimal. Because of these rapid body changes for children, self-motion information may be less well calibrated than for adults. This may explain why children used visual spatial cues to improve their visual + self-motion response variability, while adults did not. However, it does not explain why adults did not use this cue at all to reduce variability, since the visual cue was still more reliable than the self-motion. Another possibility is that children cannot help but use the visual cue heavily in spatial tasks^14^ even when the nature of the task would suggest not to do so. There is evidence that children are less selective than adults in using visual information. For example, children, but not adults, use visual information in spatial tasks, even when this cue is irrelevant (i.e., lacks validity) for the task^21^. Additionally, visual information dominates children’s audio-visual spatial judgements up to 12 years of age^14^. Our results further support this view as, although both adults and children were less influenced by the visual cue in the combined condition as a function of visual and self-motion discrepancy (as calculated by Mahalanobis distance), visual influence on the children’s performance remained more pronounced even for larger discrepancies in these cues distributions. An inability of children to discount the visual cue fits well with the recalibration theory proposed by Gori and colleagues e.g.^13^,^17^, which states that visual information is used to calibrate other modalities for spatial tasks during childhood, but not necessarily in adulthood.

Our results partly differ from those of Zhao and Warren^23^. Zhao and Warren^23^ found that the response variability of adult participants was determined by a cue combination model, while their response accuracy was determined by a cue competition model^35^. In our study, in contrast, both adults’ variable error (response variability) and constant error (response accuracy) were consistent with a cue competition model. No improvement was found when both visual and self-motion cues, rather than only visual or self-motion cue, were available during memorization. However, in terms of constant error, responses were intermediate to the two cues, suggesting that both were used over the course of the study (cue competition). This is consistent with the findings of Zhao and Warren^23^ as we also show that adults’ response accuracy was explained by a cue competition model. A cue combination model on the contrary, determined children’s response variability and accuracy. The predicted improvement in precision was found when both visual and self-motion cues were available during memorization, rather than only visual or self-motion cues alone. Our analysis suggests that the use of cue combination or competition in different groups of participants can also depend on how the same cues are experienced^23^. As the adult group’s spatial estimates were more precise (less variable), natural biases in single-cue responses would have been more detectable to adults than to children, leading adults to experience the cues as more discrepant than children. Again, this conclusion is supported by our findings that adults gave more discrepant performances in the visual and self-motion condition (when taking their precision into account), and that they switched to rely more on self-motion when this discrepancy was large.

Differences in multisensory cue combination abilities during development could reflect different maturational timescale of neural mechanisms. When given vestibular and visual information about self-motion (heading), it is now established^36^,^37^ that a sub-group of neurons in areas such as the dorsal medial superior temporal area (MSTd) respond as predicted by an optimal cue combination model, i.e., by optimally combining task to reduce uncertainty. However, another sub-group of neurons shows preference for one cue or the other, or are tuned to conflicting stimuli. How these systems cooperate to compute heading, and the general principles of neural cue integration are current topics of research – for example, “divisive normalization” has been proposed as a canonical neural computation underlying reliability-weighted averaging^38^. One recent proposal is that an altered balance of excitation and inhibition during divisive normalization could explain altered sensory processing in autism^39^. Low-level immaturities of this kind could potentially also underlie immature multisensory processing and cue combination in typically developing children. At present no study has investigated cue combination neural mechanisms for vision and active self-motion during more complex spatial tasks, and the study of these neural mechanisms during ontogenetic development is still at its infancy^40^. Future studies will be necessary to further clarify the link between children’s and adults’ cue combination and competition behavior and neuronal responses in multisensory areas.

Our results are the first to show optimal integration of visual and self-motion cues during path reproduction in childhood, thus adding essential new information. The only previous study examining optimal integration of visual and self-motion cues in both children and adults, is that of Nardini, *et al*.^9^. Nardini, *et al*.^9^ tested children up to 7–8 years, so we do not know if older children would have shown optimal integration of spatial cues at a similar age to those tested here (10–11 years old), despite the differences in spatial task. The lack of data exploring the ability of older children (>8 years old) to integrate visual and self-motion spatial representations when navigating limits our understanding of when our multisensory spatial representation develops. Although studies agree that optimal cue combination is immature at 8 years, there is not yet a clear picture of when it becomes mature, because the age at which optimal integration occurs changes with task and combination of sensory cues e.g.^13^,^14^,^15^,^16^,^40^. For example, findings from developmental studies using multisensory spatial tasks other than navigation^14^ show a lack of integration up to 12 years. This is in contrast to what we found here, since 10- to 11-year-old children did integrate, suggesting that the maturation of optimal cue integration for spatial navigation may follow specific time courses. In future, more studies could examine older children (from 10–11 to 18 years) to find at what age they do combine cues to reduce variance in different spatial tasks, and to what extent cue combination with different cues and tasks follow the same time courses. This essential knowledge would support the existence of either one common, but flexible mechanism^40^ or separate mechanisms.

Using immersive virtual reality has many advantages, but also some limitations. In HMDs the field of view is usually less than with natural vision, and the weight of the HMD can change and limit participants’ head movements, which are usually fundamental during navigation^41^. This is a general issue of virtual reality, however, here it does not influence the main findings. Participants wore the HMD at all times, when initially walking and then reproducing the path in darkness. So the effect of the HMD, if any, would have been constant, i.e., participants could move their head during reproduction as they did during memorization. Furthermore, the effect of the HMD would apply to both unimodal and bimodal memorization conditions so use of the HMD cannot explain the differences in results among the three sensory conditions. Another limitation concerns the difference between the visual task and the other conditions, in that participants in the visual task were not guided back to the starting point by following a variable and irregular path. This added step to the task for the other two conditions could have interfered with the participants’ memory of the path thus making these conditions more difficult than the visual. If this is the case, then variable and constant error for condition V (Fig. 2) might be somewhat under-estimated. However, the children’s results, which show combined-cue (V + SM) variable error close to ideal observer (MLE) predictions, indicate that any such under-estimation must be small. Any increase to the measured V variance in Fig. 2 would also increase the MLE prediction, but it is not possible for the MLE prediction to increase appreciably without participants then achieving the impossible feat of performing better than optimal. Thus, while this minor difference between conditions might have led to some under-estimation of V error, it can at most have been very minor.

To conclude, we show that though visual landmarks and self-motion can be optimally combined to guide human navigation, their combination depends not only on the reliability of these cues but also on their accuracy (i.e., consistency with the other cue). In addition, here, as in several previous studies children relied strongly on a visual cue e.g.^14^,^21^, which adults discounted as seemingly irrelevant. We propose that although the cue combination mechanism is already available in later childhood, it is still immature, and weights spatial cues in different ways to the adult mechanism. This different weighting is explained in part by the basic properties of the cues, as described by reliability (precision) and bias (accuracy), but also possibly by their perceived validity for the task, and by a tendency to over-rely on vision. Interestingly, however, in the present situation this behaviour leads to advantages for children compared to adults. These kinds of determinants of cue combination behaviour, going beyond the basic properties of reliability and bias, may also be adaptive during childhood, as the “adaptive combination” account^34^ proposes.

## Additional Information

**How to cite this article**: Petrini, K. *et al*. How vision and self-motion combine or compete during path reproduction changes with age. *Sci. Rep.* **6**, 29163; doi: 10.1038/srep29163 (2016).

## Supplementary Material

Supplementary Information
