# Supplementary material for: How vision and self-motion combine or compete during path reproduction changes with age
Source: Sci Rep. 2016 Jul 6;6:29163. doi: 10.1038/srep29163 (PMC4933893; doi:10.1038/srep29163)
Supplement: Supplementary Information [file srep29163-s1.pdf]

How vision and self-motion combine or compete during path reproduction changes with age.

Karin Petrini [1,2*], Andrea Caradonna [3], Celia Foster [3,4], Neil Burgess [5,6], Marko Nardini [7,2]

[1] Department of Psychology, University of Bath, UK

[2] UCL Institute of Ophthalmology and Department of Psychology University of Bath, UK

[3] UCL Research Department of Neuroscience, Physiology and Pharmacology, UK

[4] Centre for Integrative Neuroscience, University of Tübingen, Germany

[5] UCL Institute of Cognitive Neuroscience

[6] University College London Institute of Neurology, UK

[7] Department of Psychology, Durham University, UK

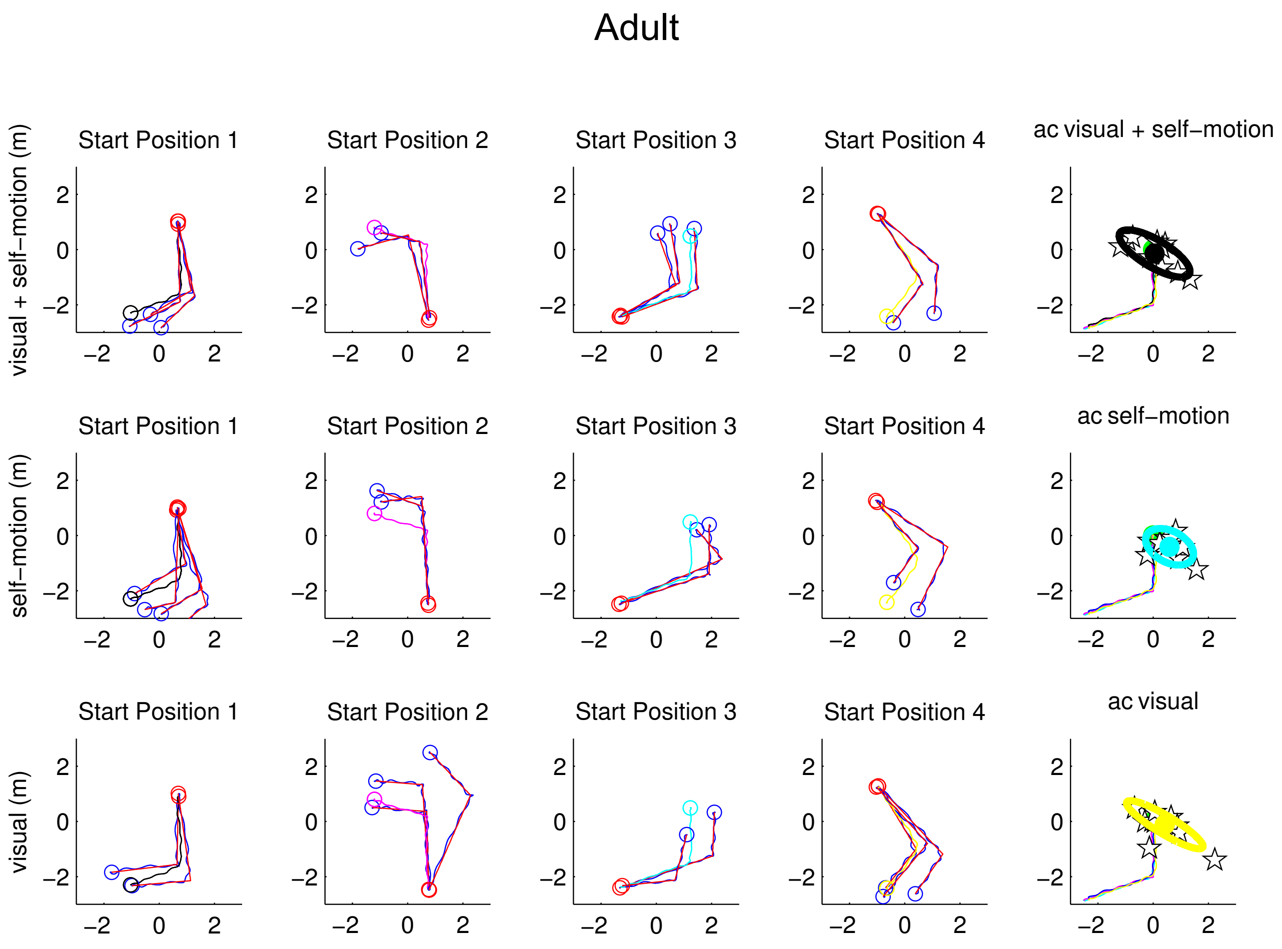

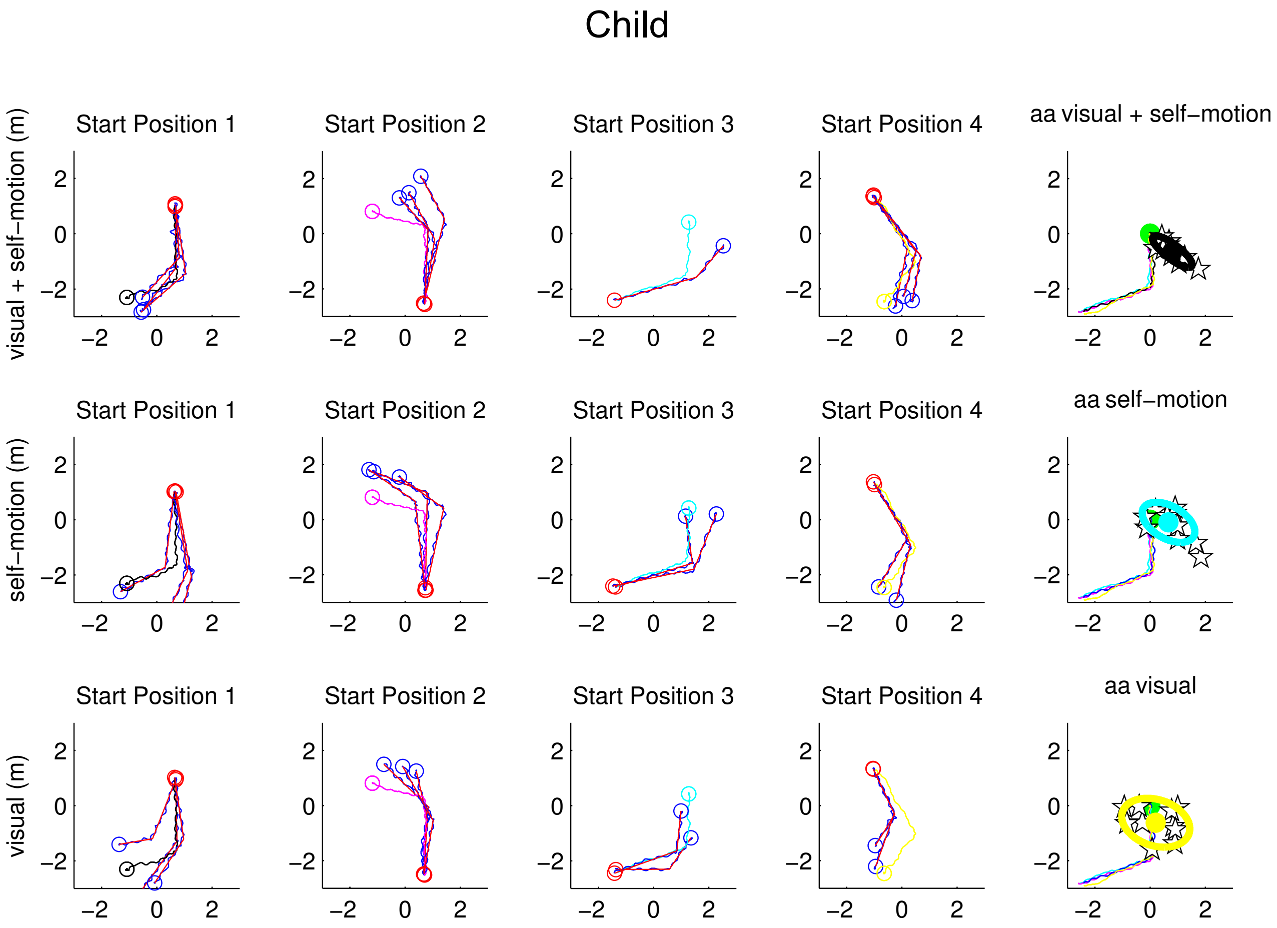

Fig. S1. Data normalization and individual estimation of variable and constant error
a) Data from a 19 year-old adult (ac). b) Data from a 10 year-old child (aa). The four diagrams from left plot the two-legged trajectory of the participant (in blue) separately for each start point against the correct trajectory (in black for start position 1, in magenta for start position 2, in cyan for start position 3, and in yellow for start position 4). The red circles indicate the start position of the participant at each trial and thus overlap within each start position, while the blue circle indicate the end point chosen by the participant (where the participant stopped after reproducing the path) for each trial. The red line fitting the participant trajectory (in blue) represent the broken stick algorithm that minimizes the square error, used to find the measures of turning point and walking distance for the 1st leg and 2nd leg of the path. The data for the four different start points were normalized using the correct end point as anchor, were rotated to align to the correct trajectory, and then were flipped to align the conditions with turn to the left to those with turn to the right. The resulting diagram is represented to the right, and plots the data for the ten trials and the ellipse used to estimate the bivariate measure of end points dispersion. The top panels represent the data for the visuo-motor condition (black ellipse), the middle panels the data for the motor condition (cyan ellipse), and the bottom panels for the visual condition (yellow ellipse). The constant error was estimated as a distance between the correct end point (green dot) and the participants' end point positions (stars).

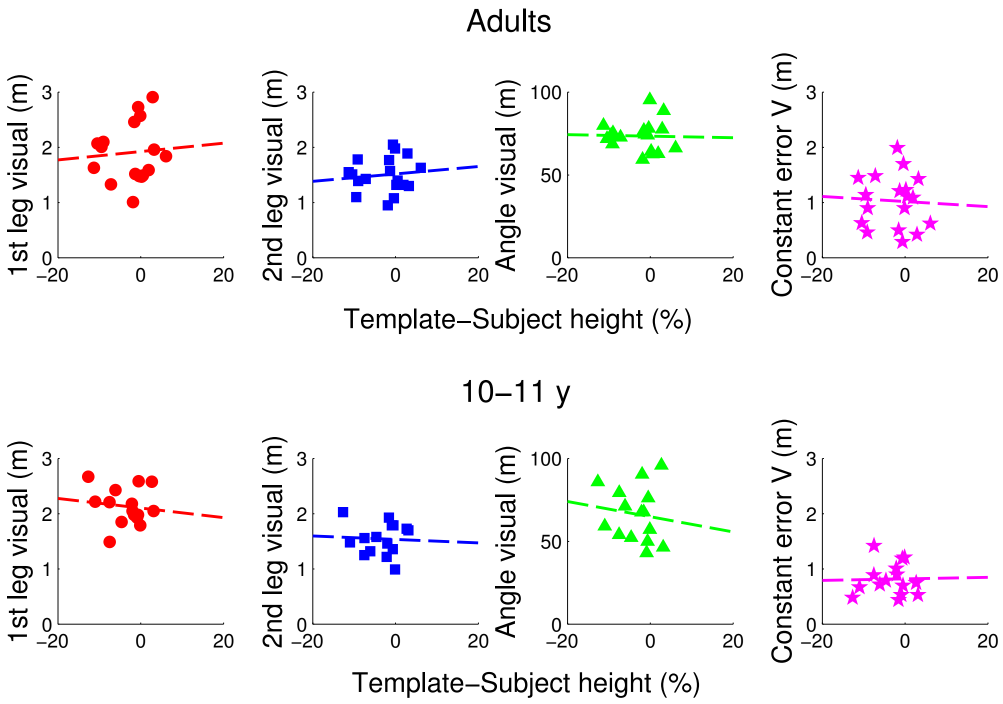

Fig. 2S. Relation between template to participant height difference and walking distance for the 1st leg of the path, 2nd leg of the path, turned angle and constant error
Moving from left to right the diagram represent the absence of a linear relation between the template to participant height difference and the distance walked for the 1st leg of the path, for the 2nd leg of the path, the turned angle, and the constant error, respectively. The top panels represent the data for the adults group, while the bottom panels for the children group.

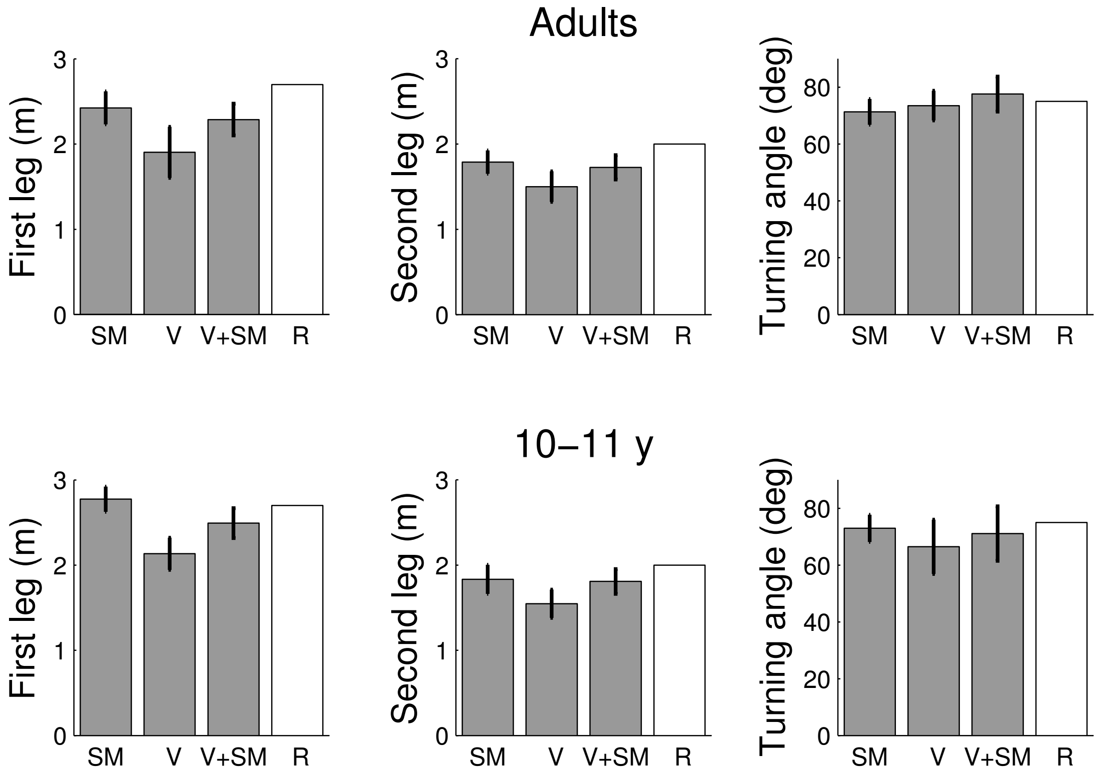

Fig. 3S. Averaged walking distance and turned angle for child and adult participants
Average results for the child and adult participants performing the motor-only (M), visual-only (V) and visuo-motor condition (VM). Moving from the left to the right the diagrams represent participants walked distance for the 1st leg of the path, for the 2nd leg of the path, and participants turned angle for the three sensory conditions (grey bars). The white bars in each diagram represent the real (R) distance or angle. Children's results are shown in the bottom panels, while adults' data are presented in the top panels. Error bars represent the standard error of the mean.
